# Effectiveness of gastrodin for migraine: A meta-analysis

**DOI:** 10.3389/fneur.2022.939401

**Published:** 2022-08-25

**Authors:** Xiu Zhou, Jingyi Shao, Xiuzhen Xie, Yingqi Xu, Tianyu Shao, Zhuqing Jin

**Affiliations:** ^1^The First School of Clinical Medicine, Zhejiang Chinese Medical University, Hangzhou, China; ^2^Dongzhimen Hospital Beijing University of Chinese Medical, Beijing, China; ^3^The Third School of Clinical Medicine (School of Rehabilitation Medicine), Hangzhou, China; ^4^School of Basic Medical Sciences, Zhejiang Chinese Medical University, Hangzhou, China

**Keywords:** gastrodin, migraine, randomized controlled trial, meta-analysis, clinical efficacy

## Abstract

**Background:**

Gastrodia elata Blume (GEB), a traditional Chinese medicine, has been widely used to treat dizziness, numbness of limbs, and infantile convulsion, among other issues. Gastrodin is the main component of GEB. This meta-analysis aimed to evaluate the efficacy and safety of gastrodin in the treatment of migraine.

**Methods:**

Ten electronic databases, namely the Cochrane Library, Embase, EBSCO, PubMed, Web of Science, CENTRAL, CNKI (China National Knowledge Infrastructure), CBM (Chinese Biomedicine Database), WanFang, and VIP (Chinese Scientific Journals Database), were searched for randomized controlled trials (RCTs) of gastrodin for migraine published before September 2021. The data were analyzed by RevMan 5.3 software and evaluated by GRADEpro.

**Results:**

A total of 1,332 subjects were included in 16 RCTs. The meta-analysis showed that gastrodin was significantly effective in treating migraine (RR = 1.21, 95%CI = [1.17, 1.27]), reducing the pain degree (MD = −1.65, 95% CI = [−2.28, −1.02]), reducing the frequency of migraine attack (SMD = −2.77, 95% CI = [−3.92, −1.62]), shortening the duration of migraine attack (SMD = −1.64, 95% CI = [−2.35, −0.93]), and slowing average arterial cerebral blood flow velocity (SMD = −3.19, 95% CI = [−5.21, −1.17]), as well as being safe.

**Conclusions:**

This systematic review revealed gastrodin is effective and safe in the treatment of migraine.

**Systematic review registration:**

https://www.crd.york.ac.uk/prospero/display_record.php?RecordID=197094, identifier: CRD42020197094.

## Introduction

Migraine is a chronic and disabling condition, and is the second most prevalent disabling condition in the world (1). It is characterized by recurrent moderate or even severe headaches lasting 4–72 h, affecting 15% of the world‘s population (2). Migraine, which seriously affects people's normal lives, is now classified by the World Health Organization as one of the 21 most serious neurological conditions (3). Gastrodia elata is a commonly used drug for the treatment of brain nervous system conditions in China. It has a wide range of applications for issues such as neurasthenia, insomnia, dizziness, and epilepsy, aspects (2), and has been included in the “Chinese Pharmacopoeia” Volume I, page 59, 2020. Gastrodin, one of the main bioactive components of GEB, is a phenolic glycoside of 4-hydroxybenzyl alcohol and selected as one of the standard compounds for evaluating the quality of GEB. Zheng et al. (4) speculated that gastrodin may be the main active ingredient in Gastrodia elata preparation, which can be used to prevent and improve migraine attacks in rats, by discussing the effects of the active ingredients of Gastrodia elata preparation on the expression of calcitonin gene-related peptide and adenosine A1 receptor in migraine model rats. After being absorbed into the blood, gastrodin is widely distributed in various tissues of rats and can enter the brain through the blood-brain barrier (5). Due to its extensive pharmacological effects and unique mechanism, it has been made into various preparations (6).

At present, the commonly used clinical drugs for migraine treatment mainly involve non-steroidal anti-inflammatory drugs (NSAIDs), opioids, and triptans. Since 2012, the conventional therapy for migraine has changed little (7). However, patients with symptomatic peripheral, coronary, and cerebrovascular diseases and severe hypertension history were forbidden to use triptans drugs. In addition, NSAIDs may induce gastrointestinal and cardiovascular diseases. Moreover, the frequent use of opioids may lead to medication-overuse headache (MOH), and also have the risk of addiction (8). Invalid treatment and drug abuse in the acute phase of migraine can worsen the condition from acute to chronic (9), causing greater burden to patients and society. Traditional Chinese medicine is a good choice, because migraine has tolerance and limited adverse reactions to supplements and substitute drugs. Acute toxicity tests showed that gastrodin and its metabolites (p-hydroxybenzaldehyde or p-hydroxybenzyl alcohol) were safe, and gastrodin preparations have been widely used in the clinical treatment of migraine. However, there is still insufficient evidence to evaluate the outcome of gastrodin for the treatment of migraine. This meta-analysis therefore aims to systematically integrate these clinical trials to assess the efficacy and safety of gastrodin, providing an evidence-based basis for further clinical use and research for curing migraines.

## Materials and methods

### Data sources and search strategy

Ten electronic databases, namely CNKI (China National Knowledge Infrastructure), CBM (Chinese Biomedicine Database), VIP (Chinese Scientific Journals Database), Wanfang, Web of Science, pubmed, EBSCO, CENTRAL, the Cochrane Library, and Embase, were searched for randomized controlled trials (RCTs) on gastrodin or gastrodin combined with other drugs for migraine. The search period ranged to June 2022. The following keywords were used for our search: gastrodin AND (migraine OR hemicrania OR megrim).

### Eligibility criteria

Inclusion criteria were set as follows: (1) RCTs; (2) the subjects were migraine patients, all of whom met the relevant diagnostic criteria of the international classification of headache diseases; (3) treatment groups used gastrodin combined with routine medication, while the control group used the same routine medication alone as the treatment group; (4) there was no significant difference in gender or age between the two groups of patients in the control group; and (5) data published in English and Chinese.

Exclusion criteria were set as follows: (1) there was no proper control group; (2) the migraine studied was as a complication of other diseases and not the main condition studied; (3) the subjects are special groups, such as pregnant women, adolescents, and children; (4) interventions using gastrodin were combined with other therapies or drugs such as acupuncture, psychotherapy, or nourishing serum brain particles that are not conventional treatments; (5) the observation indicators and observation methods were not consistent, such as using the HIT-6 score for pain scores; and (6) data were insufficient or in doubt.

### Interventions

The treatment group used gastrodin (no limitation on dosage) combined with conventional treatment for migraine; the control group used conventional treatment for migraine alone: calcium ion antagonists, calcium channel blockers, antiepileptic drugs, vasodilators, Painkillers, etc.

### Efficacy evaluation index

Clinical efficacy, pain score, frequency of headache, duration of headache, average blood flow velocity of cerebral artery, and adverse reactions were assessed.

### Data extraction

Two reviewers independently reviewed the full-text versions of all the articles retrieved in the literature search to identify eligible studies. Conflicts in study selection were resolved by a third reviewer. Firstly, reviewers strictly analyzed and selected the literature by contrasting exclusion and inclusion criteria, analyzing the literature title and abstract. Then, reviewers ensured the clinical randomized controlled trials were retained. In the end, they determined which retained literature to include after reading.

### Quality evaluation

Using the Cochrane risk of bias tool (Cochrane Handbook 5.1.0), the following aspects were assessed: random sequence generation, allocation concealment, blinding of participants and personnel, blinding of outcome assessments, incomplete outcome data, selective reporting, and other potential biases for each included study. Each domain was categorized into one of three groups: low risk, high risk, or unclear. The quality of evidence for each main outcome was evaluated by using the Grading of Recommendations Assessment, Development, and Evaluation (GRADE) approach. Using the online program GRADEpro (https://gradepro.org/), we assessed the risk of bias; inconsistency, indirectness, and imprecision of the results; and the probability of publication bias with a four-item scale (“Very Low,” “Low,” “Moderate,” or “High”). Evaluation of methodologic quality was also evaluated by two independent reviewers, who consulted with a third reviewer when there were discrepancies.

### Statistical analysis

Statistical analysis was performed on the collected data using Revman 5.3, statistical software provided by the International Evidence-Based Medicine Collaboration Network. The count data are expressed as odds ratio (OR) or relative risk (RR). RR is mainly used for cohort study; OR is mainly used in case-control studies. For continuous variables, if the measurement units and methods are inconsistent, the standardized mean difference (SMD) is used as the effect index (10). When the measurement unit and measurement method are consistent, the measurement data are expressed as mean difference (MD). Both of them are expressed as 95% confidence interval (CI). When the results showed that I^2^ < 50%, they were considered to be less heterogeneous or non-existent. When I^2^ > 50%, heterogeneity was considered to exist, indicating that the cause of heterogeneity should be analyzed by a sensitivity or subgroup analysis. In addition, sensitivity analyses were performed to identify the robustness of meta-analysis results by excluding: (1) studies with high risks of bias and (2) outliers that are numerically distant from the rest of the data. If more than ten trials were included in the meta-analysis, reporting funnel plots assessed biases such as publication bias (11).

## Meta-analysis results

### Study description

**Figure 1:**
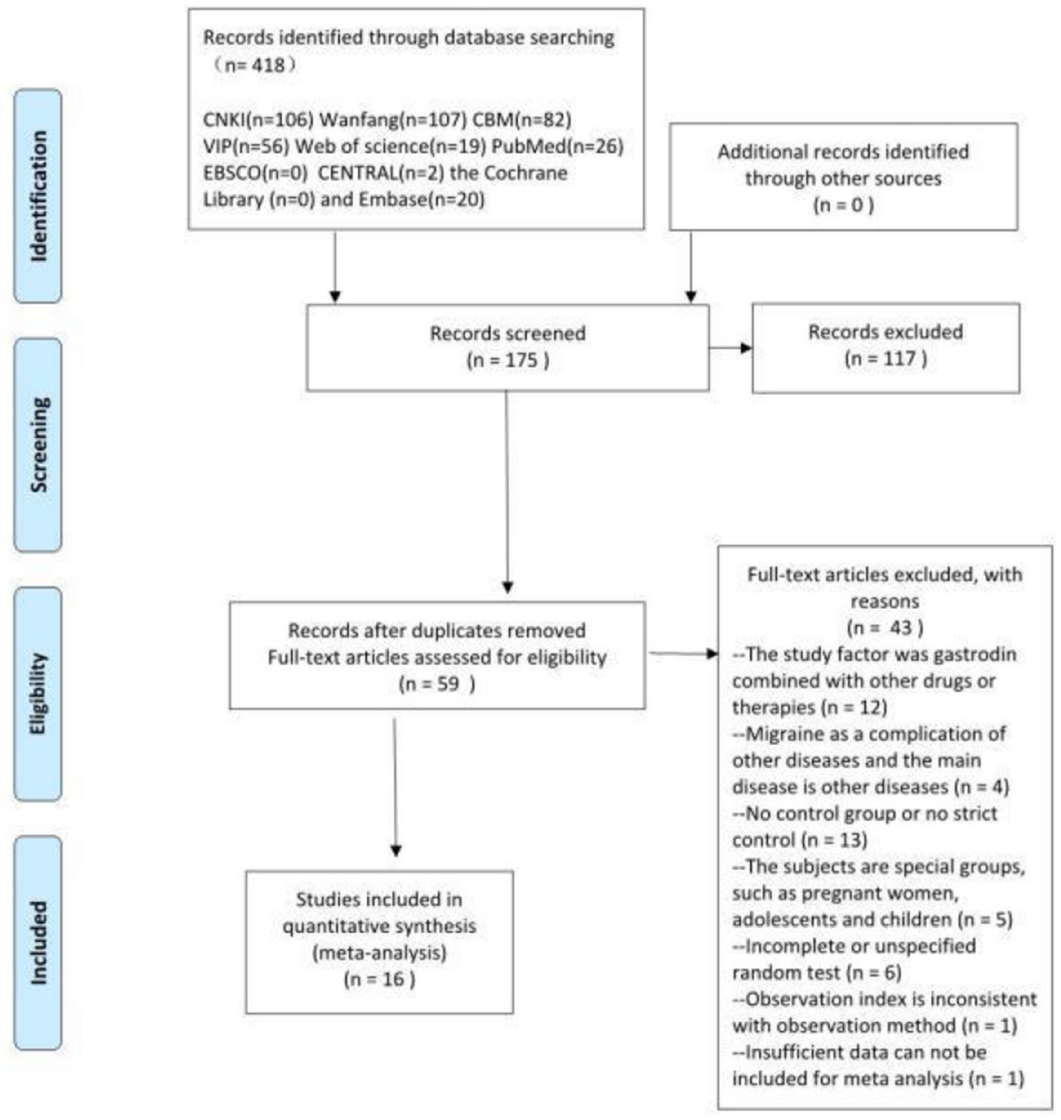
Flow chart of study selection. From Moher et al. (12).

**Figure 2:**
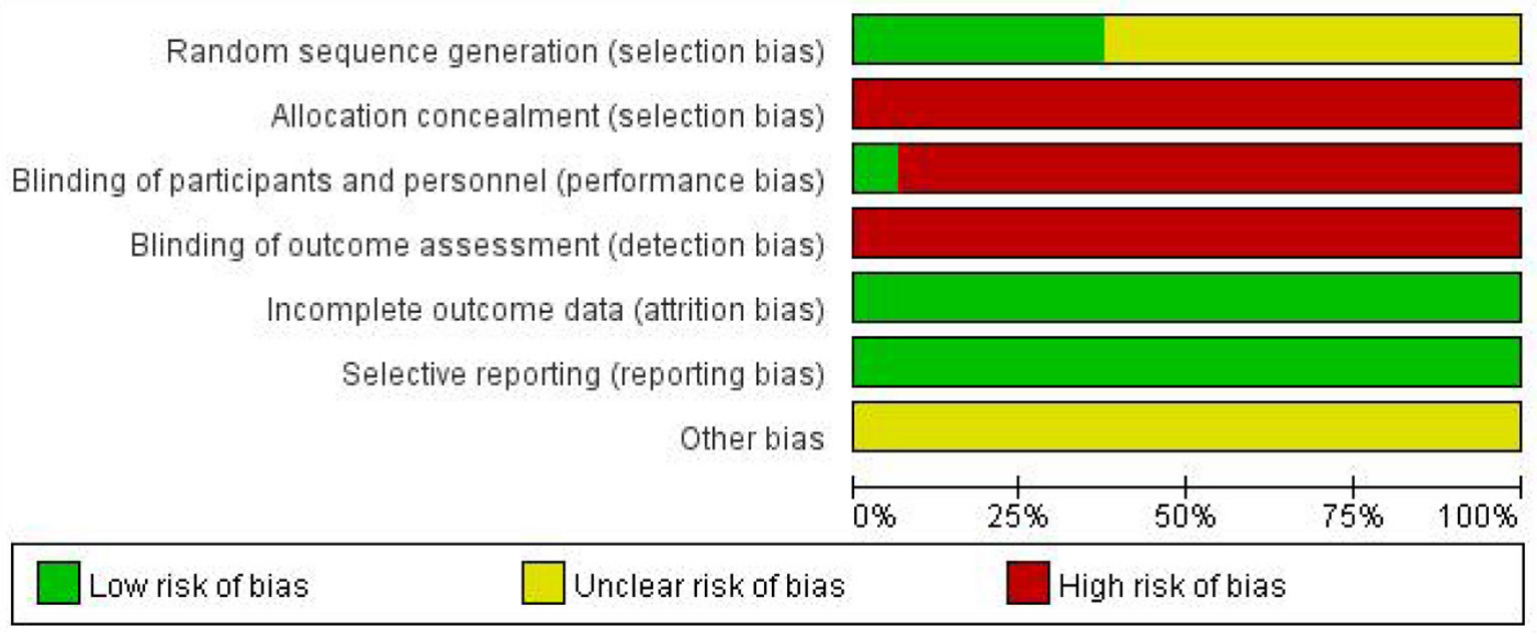
Risk of bias graph.

**Figure 3:**
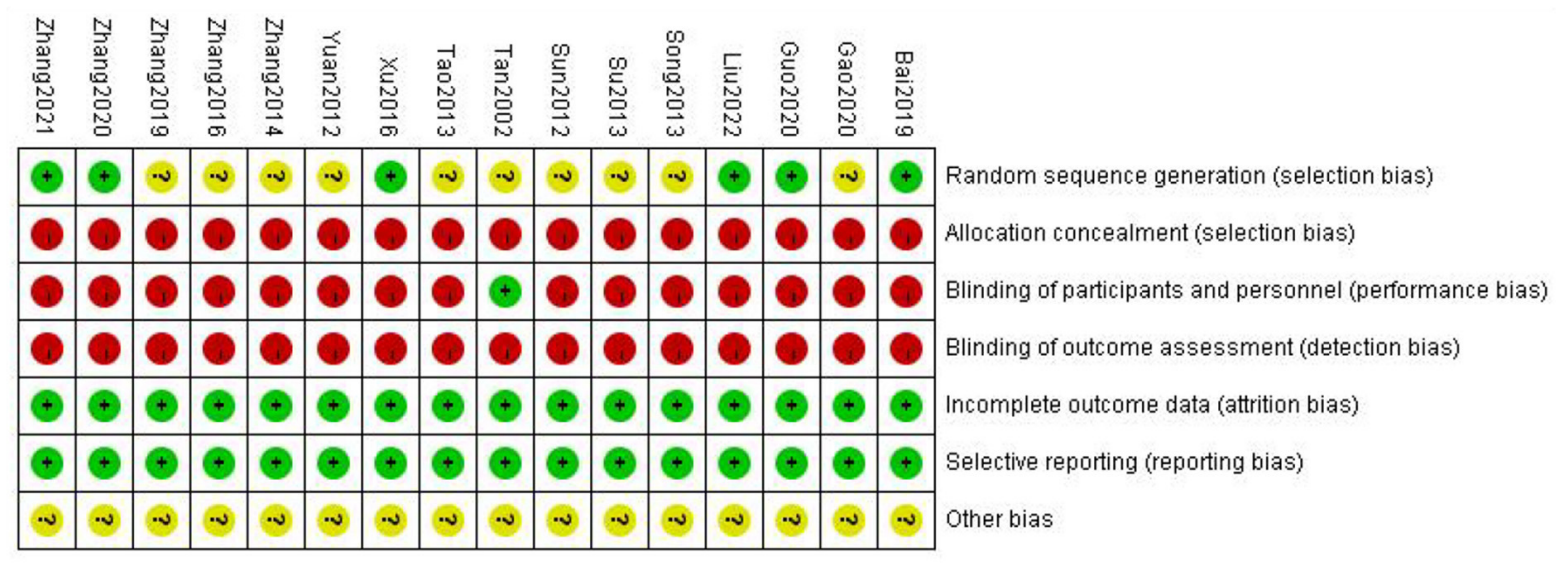
Risk of bias summary.

Based on the inclusion and exclusion criteria, 16 RCTs were ultimately included in the meta-analysis, including 1,670 participants (839 in the trial group and 831 in the control group). The sample size ranged from 40 to 240, and the treatment period ranged from 8 to 56 days. In total, 16 RCTs compared gastrodin + conventional treatment to conventional treatment alone, and one other compared gastrodin to normal saline alone. All RCTs were conducted in China. A total of 418 related papers were found based on the search strategy. 16 articles met inclusion criteria. The flowchart, the risk of bias summary, and bias graph are shown in Figures 1–3 and the basic information of the included literature is shown in Table 1. Based on Cochrane bias risk tool analysis, six studies generated random sequences by random number table method, which was considered to have low bias risk in random sequence generation. The remaining studies were considered to have a high risk of bias because they did not provide a detailed description of the random sequence generation methods. No articles mentioned allocation concealment. One study referred to double-blindness. None of the remaining studies referred to blind method. Therefore, they were evaluated to have a high bias risk. The included studies did not report withdrawal cases, and incomplete outcome data was evaluated as low bias risk.

**Table 1:** Basic characteristics of research literature included.

**References**	**Sample size (Men/Women)**	**Mean age or age range (AVG)**	**Experimental group**	**Control group**	**Treatment duration**	**Other outcomes**	**Standards for clinical efficacy appraisal**
Bai (13)	Exp:76 (40/36) Con: 76 (39/37)	Exp: 39–58 (47) Con: 38–56 (46)	Gastrodin injection + sodium valproate	Sodium valproate	2W	1 Clinical efficacy 2 Serum S-NSE 3 MMP-9	Invalid: Attack frequency decreased < by 50% or attack frequency increased compared with before treatment.
Gao and Ren (14)	Exp: 56 (26/30) Con: 56 (27/29)	Exp: 66.53 ± 19.47 Con: 66.58 ± 18.42	Gastrodin injection + dexamethasone	Dexamethasone	30D	1 Clinical efficacy 2 Duration of headache 3 Pain score 4 Frequency of headache 5 5-HT indicators 6 Adverse reactions	Invalid: the patient's symptoms were not improved, or even worse
Guo (15)	Exp: 72 (38/34) Con:72 39/33	Exp: 49.37 ± 8.14 Con: 50.15 ± 8.36	Gastrodin injection + flunarizine	Flunarizine	2W	1 Clinical efficacy 2 Vascular endothelial function 3 Nerve function	Invalid: the frequency of headache attack decreased by <50 % compared with that before treatment
Song (16)	Exp: 32 (13/19) Con: 32 (10/22)	Exp: 18–65 (42.5) Con: 20–68 (44.2)	Gastrodin injection + flunarizine	Flunarizine	2W	1 Clinical efficacy	Invalid: No significant improvement in the number of headache attacks after one course of treatment
Su et al. (17)	74 (34/40)	38.0 ± 7.6	Gastrodin injection + sibelium capsules	Sibelium capsules	1M	1 Clinical efficacy	Invalid: headache intensity reduced by <1 level, or headache duration shortened by <1/3, or headache aggravated, or headache duration prolonged
Sun (18)	Exp: 30 (11/19) Con: 30 (13/17)	Exp: 39.5 ± 8.2 Con: 39.2 ± 7.9	Gastrodin capsules + nimodipine tablet	Nimodipine tablet	15D	1 Clinical efficacy 2 Hemodynamics	Effective: −75 %; invalid: Headache relieved, attack frequency reduced <50 %.
Tan et al. (19)	40 (12/28)	15–55 (36.7)	Gastrodin injection + normal saline	Normal saline	8 D	1 Clinical efficacy 2 Qualitative indexes of spherical conjunctival circulation	Effective: headache basically or eased.
Tao and Peng (20)	Exp: 74 22/52 Con: 72 25/47	Exp: 48.53 ± 10.58 Con: 49.52 ± 11.17	Gastrodin injection + sibelium capsules	Sibelium capsules	2W	1 Clinical efficacy 2 Adverse reactions	Invalid: Symptoms and signs improved slightly after treatment, but seizure frequency did not change significantly.
Xu et al. (21)	Exp: 50 (30/20) Con: 50 33/17	Exp: 43.6 ± 25.6 Con: 52.6 ± 23.6	Gastrodin injection + sodium valproate	Sodium valproate	2 W	1 Clinical efficacy 2 Duration of headache 3 Pain score 4 Frequency of headache 5 Quality of life 6 Adverse reactions	Ineffective: After treatment, the patient's clinical symptoms and signs did not change.
Yuan (22)	Exp: 30 12/18 Con: 30 11/19	Exp: 22–68 (49.2) Con: 23–66 (48.3)	Gastrodin injection + Routine treatment of cerebrovascular dilatation drugs, Chinese patent medicine for promoting blood circulation and relieving pain	Routine treatment of cerebrovascular dilatation drugs, Chinese patent medicine for promoting blood circulation and relieving pain	1W	1 Clinical efficacy 2 Adverse reactions	Invalid: No change in headache severity, <30% fewer episodes.
Zhang (23)	Exp: 32 (13/19) Con: 26 10/16	Exp: 33.2 ± 2.1 Con: 34.3 ± 1.8	Gastrodin injection + flunarizine	Flunarizine	2 W	1 Clinical efficacy 2 Duration of headache 3 Pain score 4Frequency of headache	Invalid: No significant improvement in seizure frequency after treatment.
Zhang (24)	Exp: 41 20/21 Con: 41 22/19	Exp: 52.43 ± 1.52 Con: 52.27 ± 1.36	Gastrodin capsules+lomerizine hydrochloride capsules	Lomerizine hydrochloride capsules	8W	1 Clinical efficacy 2 Duration of headache 3 Pain score 4 Frequency of headache 5 SF-36 6 Serological indicators 7 Cerebral blood flow 8 Adverse reactions	Invalid: Headache score decreased <20% after treatment.
Zhang (25)	Exp: 120 (36/84) Con: 120 (34/86)	Exp: 50.02 ± 10.32 Con: 55.60 ± 12.35	Gastrodin injection + nimodipine tablet	Nimodipine tablet	2W	1 Clinical efficacy 2 Duration of headache 3 Pain score 4 Frequency of headache 5 Hemodynamics 6 Quality of life 7 Adverse reactions	Invalid: no significant improvement in headache, reduction rate <30%;
Zhang (26)	Exp: 35 (15/20) Con: 35 (13/22)	Exp: 60–89 (76.1) Con: 61–88 (75.3)	Gastrodin injection + Routine treatment of cerebrovascular dilatation drugs, Chinese patent medicine for promoting blood circulation and relieving pain	Routine treatment of cerebrovascular dilatation drugs, Chinese patent medicine for promoting blood circulation and relieving pain	1W	1 Clinical efficacy	Invalid: the degree of headache has not changed, and the number of attacks has been reduced by <30%
Zhang (27)	Exp: 50 (25/25) Con: 50 (24/26)	Exp: 62.56 ± 11.15 Con: 62.05 ± 11.33	Gastrodin injection + flunarizine	Flunarizine	2W	1 Clinical efficacy 2 Duration of headache 3 Pain score 4 Frequency of headache	Invalid: migraine symptoms did not alleviate after drug treatment
Liu (28)	1 68 (78/90)	51.25 ± 10.30	Gastrodin injection + nimodipine tablet	Nimodipine tablet	2W	1 Clinical efficacy 2 Duration of headache 3 Pain score 4 Frequency of headache 5 Cerebral blood flow 6 adverse reactions	invalid: The attack frequency is reduced by <30%

Exp, experimental group; Con, control group; W, weeks; D, days; M, month.

### Clinical trial efficacy analysis

**Figure 4:**
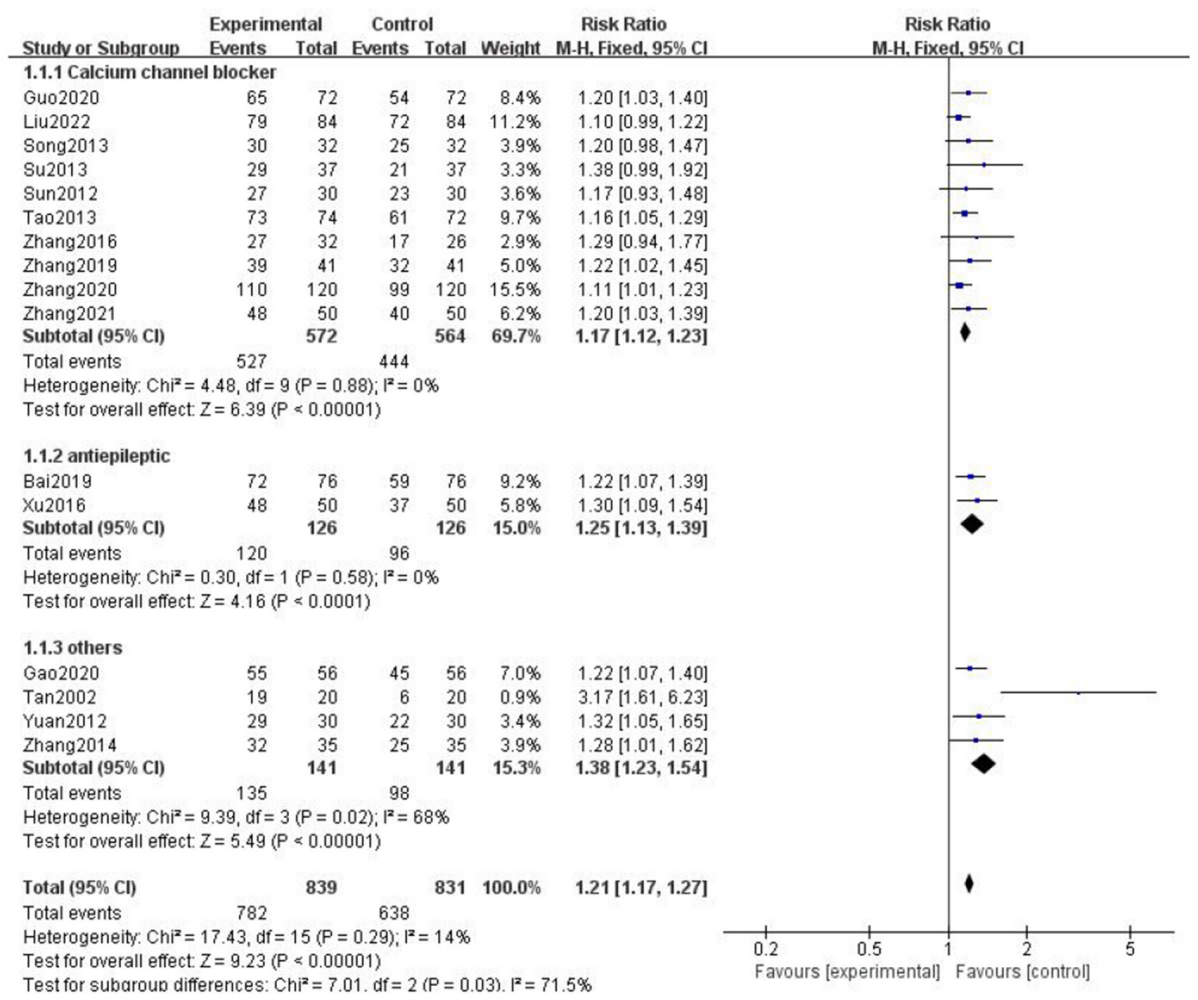
Forest chart of clinical trial efficacy divided into subgroups according to interventions.

A total of 16 studies reported the clinical efficacy of gastrodin combined with conventional treatment compared with conventional treatment alone. The subgroup analysis was performed by interventions as the grouping standard. The heterogeneity test results indicated that the interventions may be the main source of heterogeneity. The results supported that gastrodin can effectively treat migraine (Figure 4).

**Figure 5:**
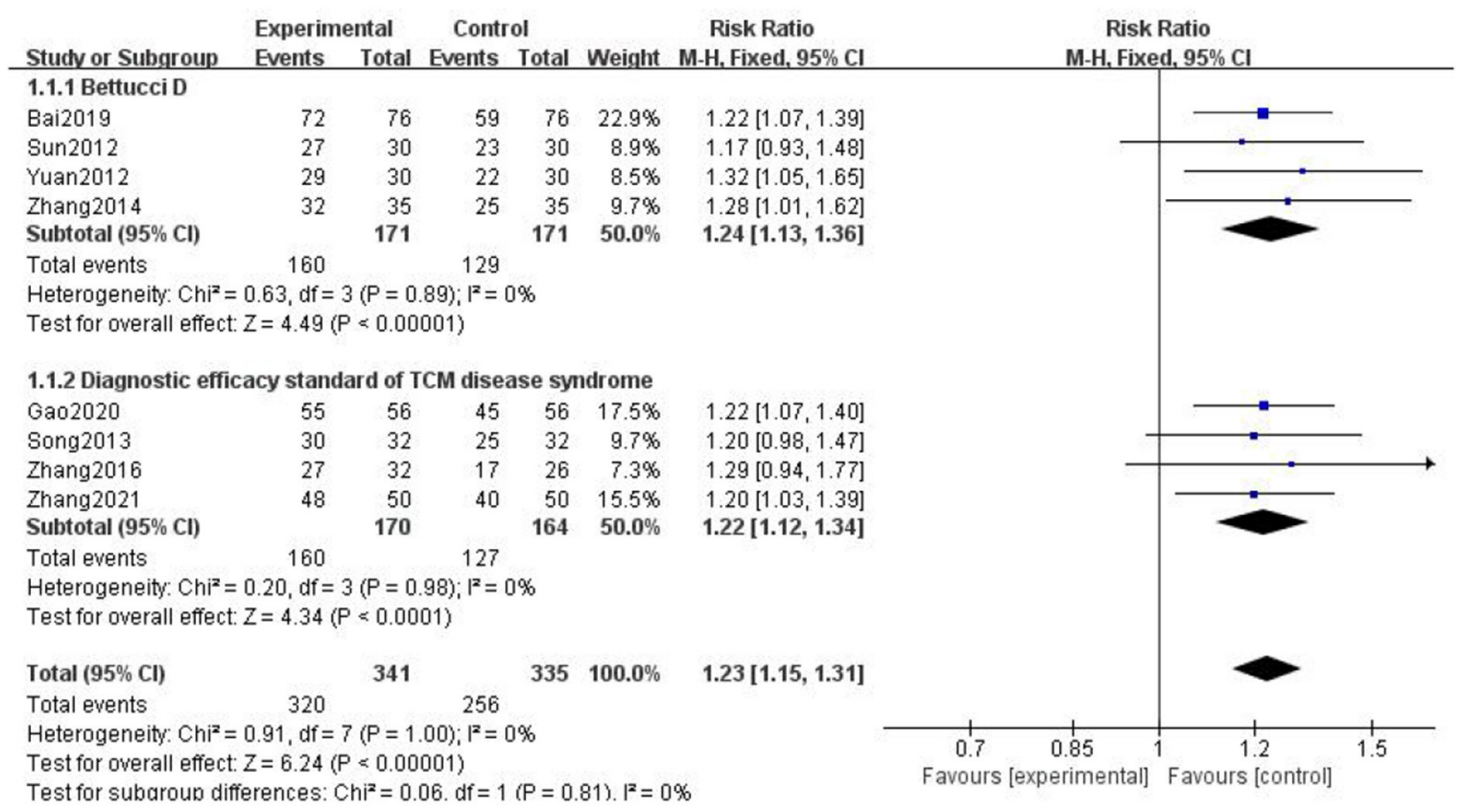
Forest chart of clinical trial efficacy divided into subgroups according to evaluation criterion.

Due to the different evaluation criteria for clinical efficiency of the included literature, we chose the more official evaluation criteria. And according to different evaluation criteria (diagnostic efficacy criteria of TCM syndromes) (29), we have classified and merged the results. The results are as follows (Figure 5).

We found that Bai (13), Sun (18), Yuan (22), and Zhang (26) adopted the evaluation criteria formulated by Bettucci et al., and the combined results showed that *p* < 0.00001, I^2^ = 0; Gao (14), Song (16), Zhang (23), and Zhang (27) adopted the efficacy standard of migraine formulated in the diagnostic efficacy standard of TCM. The combined results showed that *p* < 0.0001, I^2^ = 0. The above results showed that gastrodin combined with conventional treatment was more effective than conventional treatment alone in the treatment of migraine.

### Pain score

**Figure 6:**
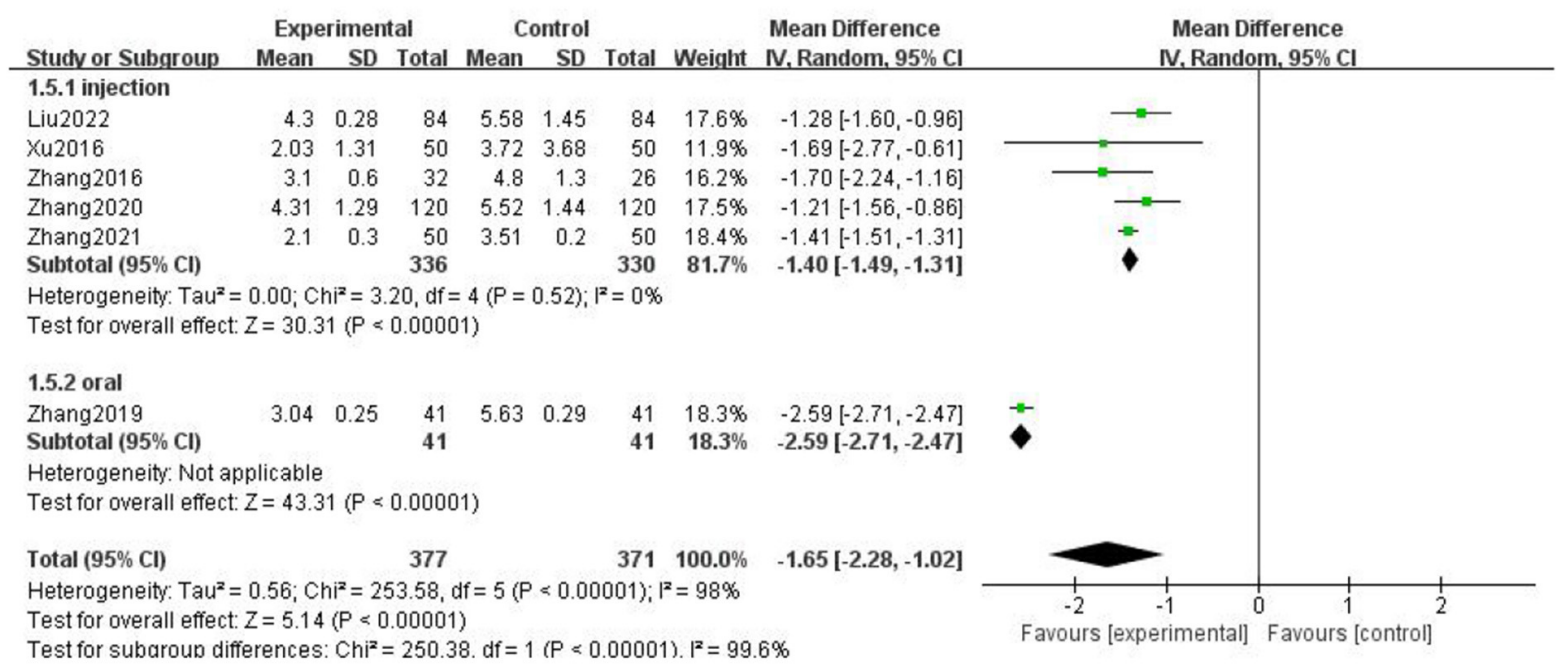
Forest chart of headache score divided into subgroups according to medication mode.

For the pain score of headache, we combined all the literature using VAS score, and selected MD for statistics. Taking the medication mode as the standard for subgroup analysis, we found that there was statistical significance in the injection group (*p* < 0.00001) and the heterogeneity decreased to 0, suggesting that the medication mode may be the main source of heterogeneity. The results showed that the experimental group using gastrodin combined with conventional treatment had significant advantages in reducing pain scores (Figure 6).

### Headache duration

**Figure 7:**
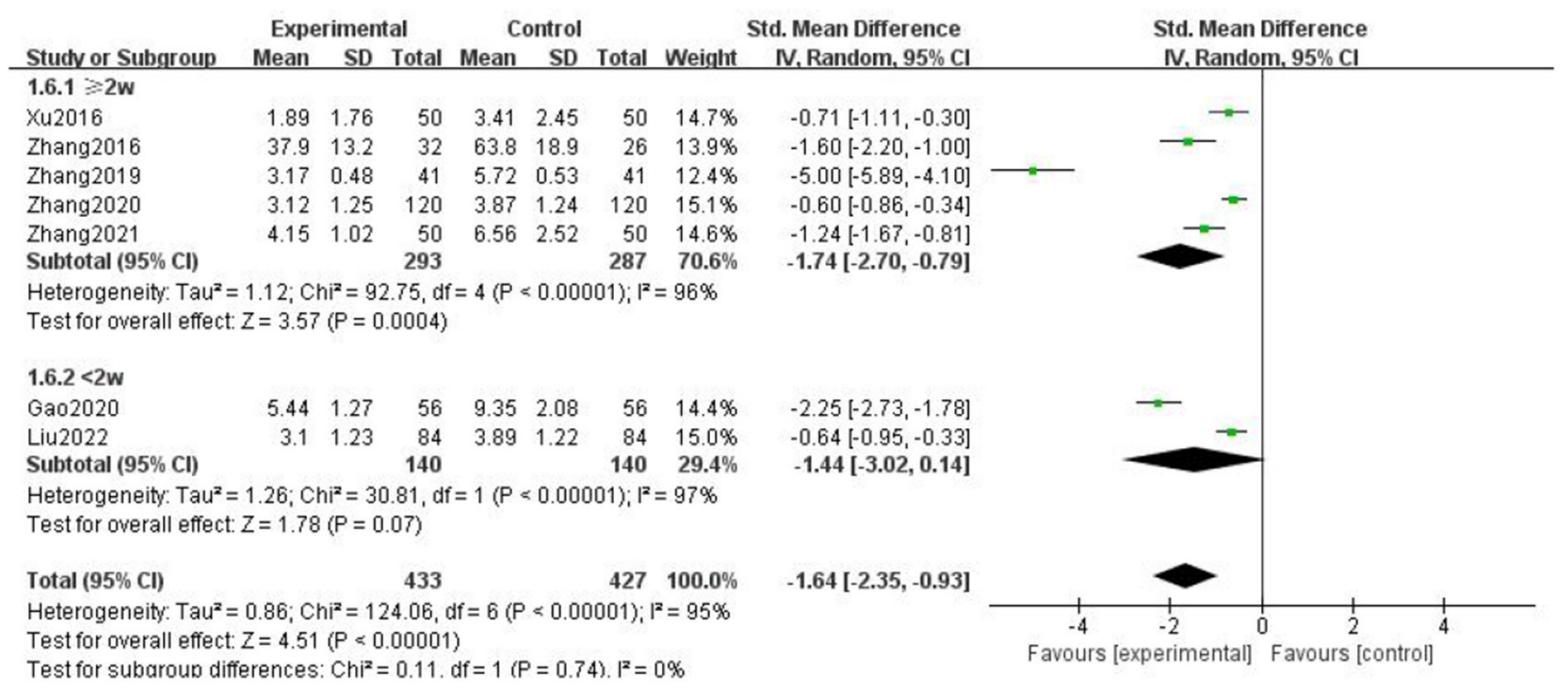
Forest chart of headache duration divided into subgroups according to course of treatment.

For the duration of headache, all literature were analyzed according to whether the course of treatment was more than 2 weeks. We found that there was no statistical difference when the course of treatment was less than 2 weeks (*p* = 0.07), and there was statistical significance in the subgroup when the course of treatment ≥2 weeks (*p* = 0.0002), but the heterogeneity was not significantly reduced, suggesting that gastrodin combined with conventional drugs can improve the duration of migraine for at least 2 weeks (Figure 7).

**Figure 8:**
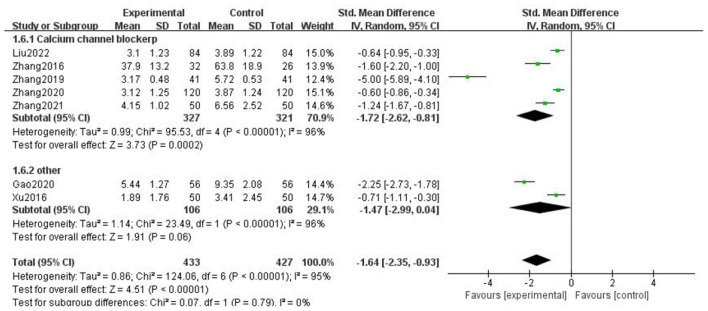
Forest chart of headache duration divided into subgroups according to interventions.

When all literature were analyzed according to whether the combined drugs were calcium channel blockers, we found that there was no statistical difference in the non-calcium channel blocker subgroup (*p* = 0.06), and there was statistical difference in the calcium channel blocker subgroup (*p* = 0.0002), but the heterogeneity was not significantly reduced. It was suggested that gastrodin combined with calcium channel blockers could shorten the duration of migraine (Figure 8).

**Figure 9:**
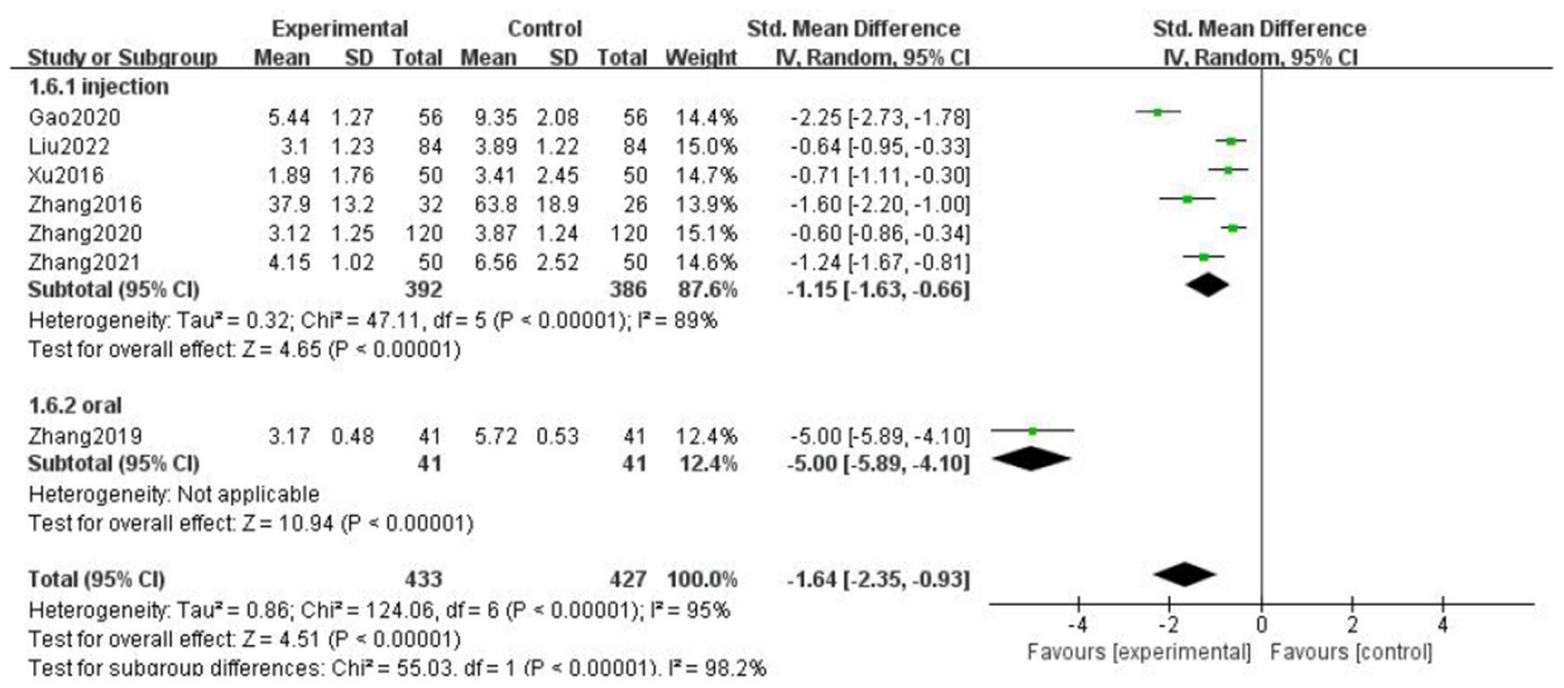
Forest chart of headache duration divided into subgroups according to medication mode.

When all the literature were analyzed according to the medication mode, we found that the heterogeneity of the two subgroups did not decrease significantly, but there were statistical differences (Figure 9).

The above results suggest that the course of treatment, the drugs combined in the intervention measures, and the medication mode are not the source of heterogeneity of headache duration. After subgroup analysis, the effective stress did not change significantly, and our research results were relatively stable.

### Frequency of headache

**Figure 10:**
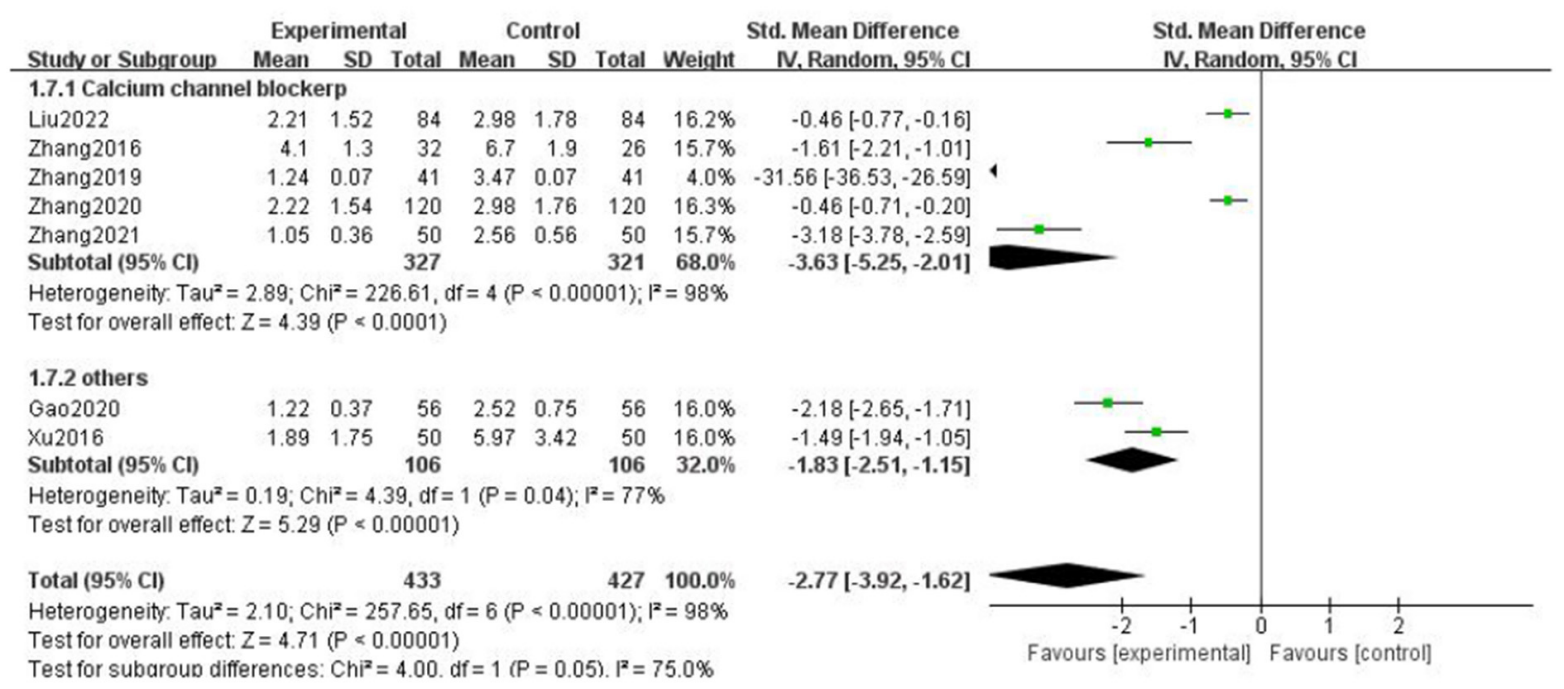
Forest chart of frequency divided into subgroups according to interventions.

**Figure 11:**
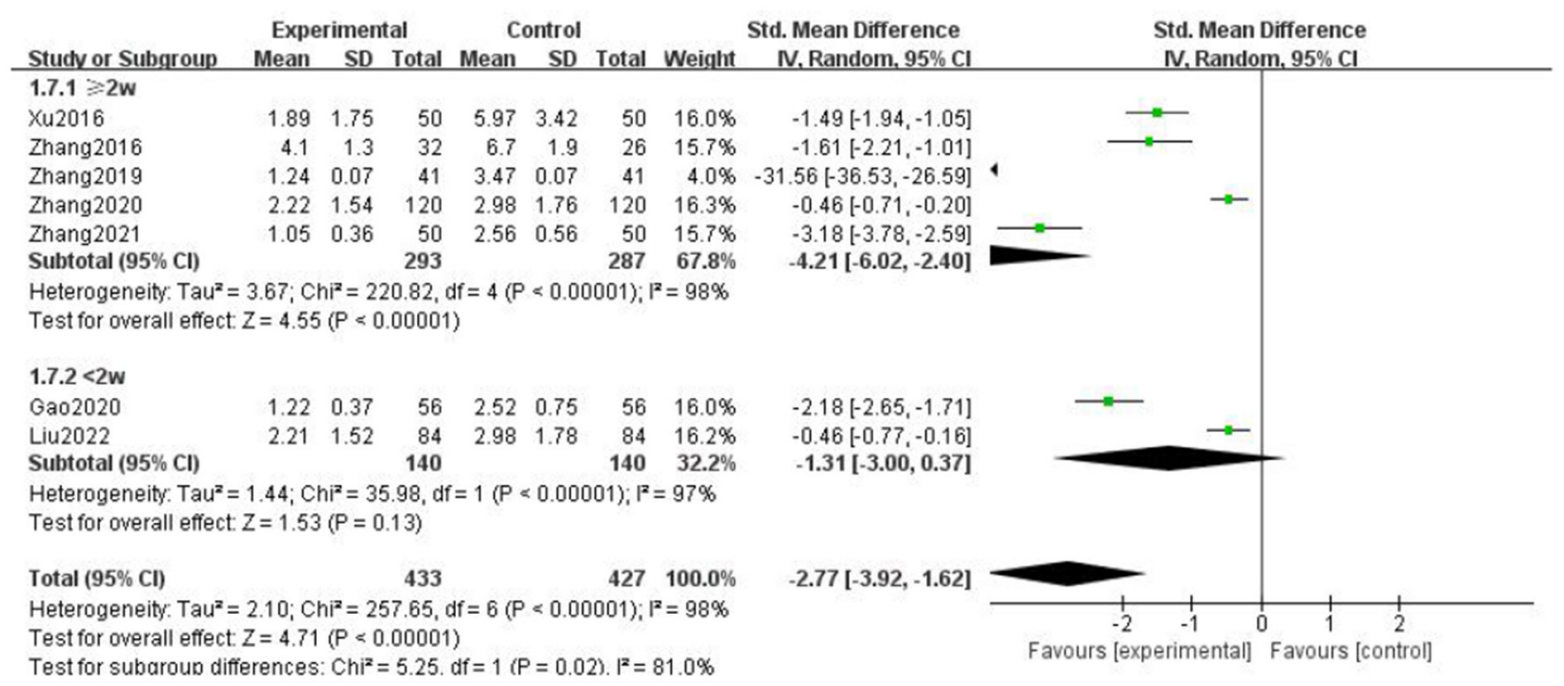
Forest chart of frequency divided into subgroups according to course of treatment.

**Figure 12:**
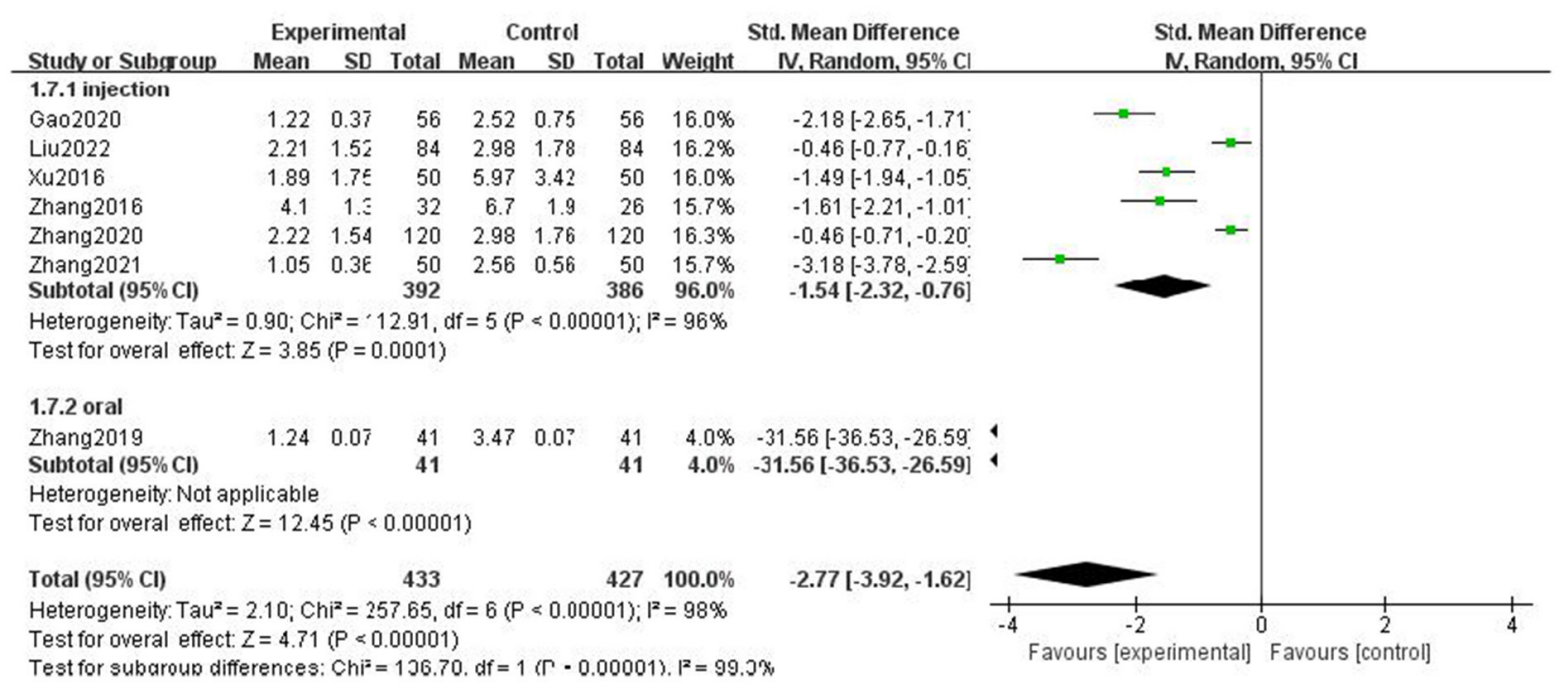
Forest chart of frequency divided into subgroups according to medication mode.

For the frequency of headache attack, we analyzed all the literature according to whether the course of treatment was more than 2 weeks, whether the type of combined drugs in the intervention measures was calcium channel blocker, and the medication mode of gastrodin. It was found that the heterogeneity of the subgroups was not significantly reduced, but there were statistical differences. The above results showed that the type of combined drugs, the course of treatment, and intervention measures were not the source of heterogeneity of headache attack frequency, there was no significant change after multifactor subgroup analysis, and our results were relatively stable (Figures 10–12).

### Cerebral artery mean blood flow velocity

**Figure 13:**
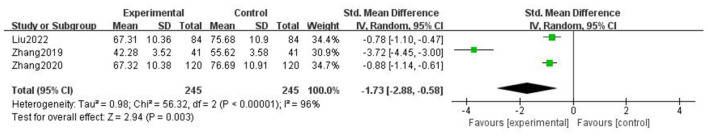
Forest chart of average blood flow velocity of MCA.

Three studies reported the average blood flow rate of cerebral arteries in the case of gastrodin combined with conventional treatment and conventional treatment alone. Some studies had demonstrated that cerebral arterial blood flow was significantly accelerated in patients with migraine and that middle cerebral artery (MCA) changes were the most obvious and common (30), so the average blood flow velocity of MCA was chosen to use. The heterogeneity test results showed that *p* = 0.003, I^2^ = 96%, indicating the literature included in the study is heterogeneous and a random effect model was used. The results showed that SMD = −1.73, 95% CI = (−2.88, −0.58), *p* < 0.00001, indicating that gastrodin combined with conventional treatment slowed down the average blood flow velocity of the middle cerebral artery in patients with migraine compared with conventional treatment alone. Through the sensitivity analysis of literature one by one, the main source of heterogeneity was Zhang (24). By reading the original text, we speculate that its sample size may be significantly different from that of the rest of the literature, so it is the main source of heterogeneity (Figure 13).

### Analysis of adverse reactions

**Figure 14:**
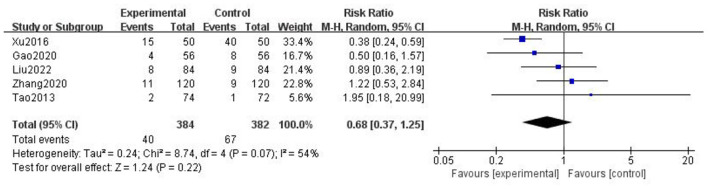
Forest chart of adverse reactions.

A total of five articles mentioned relatively significant adverse reactions (Table 2). The meta-analysis diamond was on the left side of the midline. The results showed that RR = 0.68, 95% CI = (0.37, 1.25), *p* = 0.22, but the heterogeneity was high. Through the sensitivity analysis of literature one by one, the main source of heterogeneity was Xu et al. (21) (Figure 14).

**Table 2:** Adverse reactions.

**Gao and Ren (14)**	**Tao and Peng (20)**	**Xu et al. (21)**	**Zhang et al. (25)**	**Liu (28)**
Only the adverse reaction incidence rate of vomiting, lethargy and dizziness was recorded Experimental group: 7.14%
	Experimental group: dizziness and lethargy (1 case); dry mouth and nausea (1 case)	Experimental group: dizziness (5 cases); limb numbness (2 cases); weakness (3 cases); memory decline (5 cases)	Experimental group: nausea (2 cases); dizziness (6 cases); lethargy (2 cases); diarrhea (1 case)	Experimental group: nausea (1 case); dizziness (5cases); lethargy (1 case); diarrhea (1 case)
Control group: 14.28%	Control group: lethargy and weakness (1 case)	Control group: dizziness (15 cases); limb numbness (8 cases); weakness (2 cases); memory decline (15 cases)	Control group: nausea (3 cases); dizziness (5 cases); lethargy (1 case)	Control group: nausea (2 cases); dizziness (4 cases); lethargy (2 cases)

Xu et al. (21) reported adverse reactions of dizziness, limb numbness, fatigue, and decreased memory. The incidence of adverse reactions was 30%, much lower than 80% of the control group, which indicated that gastrodin combined with conventional treatment may reduce the incidence of adverse reactions. However, this conclusion is doubtful and needs further investigation. Without the consideration of that article, the heterogeneity reduced to 0%, and there was no significant difference between two groups. In addition, most of the articles did not report obvious adverse reactions. Therefore, we can speculate that gastrodin is relatively safe.

### Publication bias

**Figure 15:**
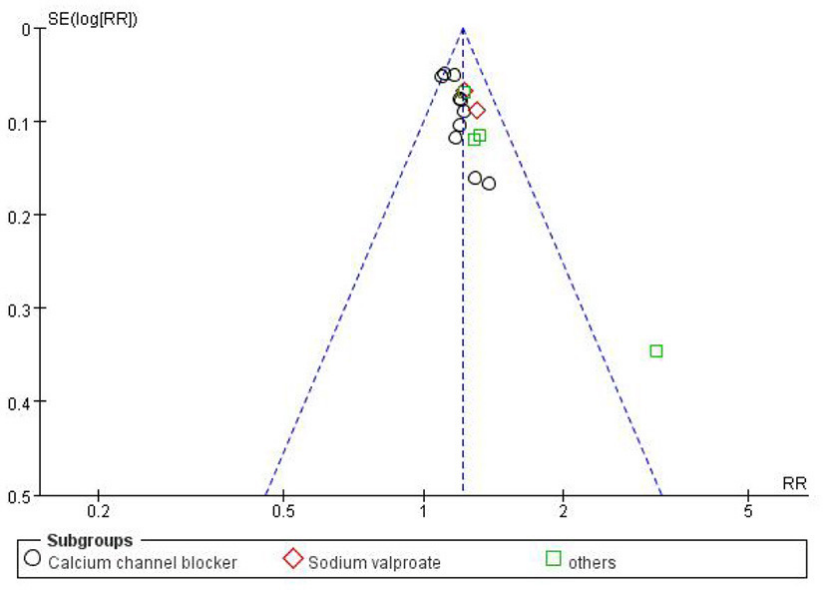
Funnel chart of clinical trial efficiency.

The clinical trials included in the literature were checked for bias, and the results were displayed in a funnel chart. The results showed a certain publication bias (Figure 15).

### Quality of evidence

In the comparison of gastrodin combined with conventional treatment for migraine and conventional treatment for migraine alone, the quality of the evidence is “very low” to “low” (Table 3). There was no high-quality evidence. The main reason for the downgrade of evidence was that the randomized controlled trials included in the meta-analysis have large limitations and large heterogeneity in each study. In addition, the sample size or number of events included in some studies is small, which limits the accuracy. Publication bias also reduces the quality of the evidence.

**Table 3:** Quality assessment.

**Quality assessment**							**No of patients**		**Effect**		**Quality**	**Importance**
**No of studies**	**Design**	**Risk of bias**	**Inconsistency**	**Indirectness**	**Imprecision**	**Other considerations**	**Gastrodin**	**Control**	**Relative (95% CI)**	**Absolute**		
**Clinical efficiency**												
16	Randomized trials	Serious^a^	No serious inconsistency	No serious indirectness	No serious imprecision	Reporting bias^b^	782/839 (93.2%)	638/831 (76.8%)	RR 1.21 (1.17 to 1.27)	161 more per 1,000 (from 131 more to 207 more)	⊕⊕○○ Low	Critical
								77.2%		162 more per 1,000 (from 131 more to 208 more)		
**VAS (better indicated by lower values)**												
6	Randomized trials	Serious^a^	Serious^c^	No serious indirectness	No serious imprecision	Reporting bias^b^	377	371	–	MD 1.65 lower (2.28–1.02 lower)	⊕○○○ Very low	Critical
**Duration (better indicated by lower values)**												
7	Randomized trials	Serious^a^	Serious^c^	No serious indirectness	No serious imprecision	Reporting bias^b^	433	427	–	SMD 1.64 lower (2.35–0.93 lower)	⊕○○○ Very low	Critical
**Frequency (better indicated by lower values)**												
7	Randomized trials	Serious^a^	Serious^c^	No serious indirectness	No serious imprecision	Reporting bias^b^	433	427	–	SMD 2.77 lower (3.92–1.62 lower)	⊕○○○ Very low	Critical
**MCA (better indicated by lower values)**												
3	Randomized trials	Serious^a^	Serious^c^	No serious indirectness	Serious^d^	Reporting bias^b^	245	245	–	SMD 1.73 lower (2.88–0.58 lower)	⊕○○○ Very low	Critical
**Adverse reactions**												
5	Randomized trials	Serious^a^	No serious inconsistency	No serious indirectness	No serious imprecision	Reporting bias^b^	40/384 (10.4%)	67/382 (17.5%)	RR 0.68 (0.37–1.25)	56 fewer per 1000 (from 110 fewer to 44 more)	⊕⊕○○ Low	Important
								10.7%		34 fewer per 1,000 (from 67 fewer to 27 more)		

a Absence of description of blindness and randomization.

b Funnel asymmetry.

c Heterogeneity.

d Events < 3.

## Discussion

In this study, the efficacy of gastrodin combined with Western medicine for the treatment of migraine was analyzed for the first time. The results showed that gastrodin combined with conventional therapy had better clinical efficacy than conventional therapy alone, and could more effectively alleviate the degree of pain, reduce the frequency of attack, shorten the attack time, and slow down the average blood flow velocity of cerebral artery. The included clinical studies showed that gastrodin treatment could reduce the levels of serum high-sensitivity C-reactive protein (hs-CRP), homocysteine (Hcy), high lipoprotein a (LPA), vascular endothelial growth factor (VEGF), substance P (SP), serum neuron-specific enolase (S-NSE), matrix metalloproteinases-9 (MMP-9), endothelin-1 (ET-1), nitric oxide (NO), calcitonin gene-related enzyme (CGRP), serum brain-derived neurotrophic factor (BDNF), and 5-hydroxytryptamine (5-HT). Significantly lower middle cerebral artery, anterior cerebral artery, posterior cerebral artery, basilar artery, vertebral artery blood flow velocity, and improved patient quality of life score play a role in the treatment of migraine.

In a variety of pathogenesis of migraine, the trigeminal nerve vascular theory dominates. The expansion and inflammation of the meningeal vessels make the trigeminal nerve release neurotransmitters such as CGRP, thereby activating the trigeminal nerve vascular system and causing pain (31). Zheng et al. proposed that gastrodin in the effective components of Gastrodia elata preparations could inhibit the occurrence of migraine by restaining the expression of calcitonin gene-related peptide (4). Neurogenic inflammation is reduced, thereby alleviating pain, lowering seizure frequency, and shortening seizure time (32, 33). Due to changes in biochemical factors and autonomic nervous system dysfunction, the middle cerebral artery and basilar artery blood flow velocity of migraine patients were significantly faster than the control group. Modern pharmacological studies have shown that gastrodin has the effects of sedation, analgesia, lowering blood pressure, and improving oxygen supply capacity (34), which can slow down the average blood flow velocity of cerebral artery and alleviate the attack of migraine (25).

In summary, gastrodin treatment of migraine can reduce serum concentration of biochemical factors which induced migraine (ET, CGRP, β-EP, SP, NO, etc.) and reduce cerebral artery blood pressure, slow cerebral artery blood flow, and improve cerebral artery oxygen supply capacity.

There are certain limitations to this study. The number of included studies is limited, and more standardized, rigorous, and large-sample high-quality studies are needed to provide more evidence for the clinical application of gastrodin in the treatment of migraine. This study confirmed the role of gastrodin in the treatment of migraine, hoping to provide a feasible alternative treatment for patients with unsatisfactory conventional treatment, or those who are unable to maintain the original treatment due to drug intolerance, drug dependence, and adverse reactions.

## Conclusions

In summary, gastrodin is effective and safe in the treatment of migraine. More rigorous long-term follow-up randomized, controlled, double-blind, large-scale trials are needed to confirm the results of this meta-analysis. This study may help guide the rational use of gastrodin and the design of future clinical trials.

## Data availability statement

The original contributions presented in the study are included in the article/supplementary material, further inquiries can be directed to the corresponding author.

## Author contributions

XZ and ZJ designed the research study. XZ and XX performed the research. JS and TS provided help and advice. XZ analyzed the data. XZ, XX, and YX wrote the manuscript. All authors contributed to editorial changes in the manuscript. All authors read and approved the final manuscript.

## Funding

This study was supported by the National Natural Science Foundation of China (No. 81774010) and Zhejiang Natural Science Foundation Project (No. ly14h270013).

## Conflict of interest

The authors declare that the research was conducted in the absence of any commercial or financial relationships that could be construed as a potential conflict of interest.

## Publisher's note

All claims expressed in this article are solely those of the authors and do not necessarily represent those of their affiliated organizations, or those of the publisher, the editors and the reviewers. Any product that may be evaluated in this article, or claim that may be made by its manufacturer, is not guaranteed or endorsed by the publisher.

## Abbreviations

GEB: Gastrodia elata Blume
RCT: randomized controlled trial
OR: odds ratio
RR: risk ratio
MD: mean difference
SMD: standardized mean difference
CI: confidence interval
NSAIDs: non-steroidal anti-inflammatory drugs
MCA: middle cerebral artery
hs-CRP: high-sensitivity C-reactive protein
Hcy: homocysteine
LPA: high lipoprotein a
VEGF: vascular endothelial growth factor
SP: substance P
S-NSE: serum neuron-specific enolase
MMP-9: matrix metalloproteinases-9
ET: endothelin
CGRP: calcitonin gene-related enzyme
BDNF: brain-derived neurotrophic factor
5 - HT: 5-hydroxytryptamine
β-EP: β-endorphin
NO: nitric oxide.
